# What Does Physiological Mean?

**DOI:** 10.1093/function/zqad042

**Published:** 2023-08-04

**Authors:** Ole H Petersen

**Affiliations:** School of Biosciences, Sir Martin Evans Building, Cardiff University, Wales CF10 3AX, UK

**Keywords:** physiological conditions, in vivo, immune cells, epithelial cells, neurones, astrocytes

I recently reviewed an original paper with a title including the words “physiological stimulation.” In this particular case, it turned out that the stimulation was far from physiological. The concentration of the hormone used for activation was way above the maximal level ever observed in vivo, and this made me think about the use, and misuse, of the word “physiological.” It is a word that we (physiologists) employ frequently and perhaps too frequently. Papers in physiological, and other, journals often refer to “physiological conditions,” which sometimes is taken to indicate experiments in vivo, but also frequently just means that experiments on single cells or tissue fragments were carried out with stimulation protocols and under circumstances that are not unlike those that could happen in vivo.

We have lived through a long and productive period of single-cell biology. Very important discoveries of real significance have been made, but it is now becoming increasingly clear that there are many critically important interactions between different adjacent cell types in most tissues. To characterize these processes, it is necessary to observe simultaneously more than one cell type in individual organs or tissues. Furthermore, the behavior of a particular cell type in isolation may not be the same as when it is embedded in its normal environment. Of particular concern is the tacit assumption in many studies that processes in cell lines reflect those in normal cells in situ. It may therefore be useful to reflect on the usefulness of working under real physiological conditions, notwithstanding the obvious difficulties of doing so. In what follows, I’ll try to illuminate these issues by examples from immunology, epithelial physiology, and neuroscience.

Ca^2+^ signaling studies in immune cells have been immensely successful in unravelling key Ca^2+^ transport events and, in particular, the properties of the Ca^2+^ release activated Ca^2+^ (CRAC) channel of the Orai type and its molecular control mechanism.^1^ Unlike the situation in epithelial cells, where Ca^2+^ signaling studies have largely been conducted on normal cells,^2^ very many studies on immune cells have been conducted on cell lines and have focused on defining molecular pathways rather than describing physiological signaling patterns. The studies of Markus Hoth and collaborators have, however, revealed important aspects of physiological Ca^2+^ signaling.^3,4^ One critical element of the physiology of T cell activation is the role of the immunological synapse.^4^ This is a specialized junction between a T cell and an antigen-presenting cell that establishes efficient communication between the cells, thereby promoting activation of the immune response. The generation of the immunological synapse focuses the Ca^2+^ signaling domain at the inner mouth of the CRAC/Orai1 channel by translocating the mitochondria close to interaction points between the CRAC channels and the endoplasmic reticulum Ca^2+^ sensor STIM1. The critical role of the immunological synapse dictates that the T cell becomes functionally polarized and, like epithelial cells,^2^ can generate both local and global Ca^2+^ signals.^3,4^ The immunological synapse allows the creation of large Ca^2+^ signals locally, immediately below the plasma membrane, in such a way that the hotspot of Ca^2+^ entry occurs at some distance from the site of the Ca^2+^ pumps in the plasma membrane, preventing immediate expulsion of Ca^2+^ entering through the CRAC channels.^4^

Ca^2+^ signaling has recently been studied in normal macrophages embedded in the exocrine pancreatic tissue.^5^ These endogenous immune cells display oscillatory Ca^2+^ signals in response to activation of various purinergic receptors, but do not normally respond to stimulation with IgG. However, after induction of alcohol-related pancreatitis, when there is major recruitment of macrophages into the pancreas, these cells generate repetitive Ca^2+^ spikes when challenged in this way.^5^ In pancreatitis, many products, including ADP and ATP from dying acinar cells, appear in the immediate environment of the endogenous macrophages, activating these cells.^5^

In epithelia, and particularly exocrine gland epithelia, there has been a long tradition for Ca^2+^ signaling studies in normal cells.^2^ Such studies gave rise to the concept of Ca^2+^ signal generation by primary release of Ca^2+^ from intracellular stores^2,5^ and later to the identification of IP_3_ as an intracellular messenger.^2^ Furthermore, the important concept of physiological local Ca^2+^ signaling and pathological global Ca^2+^ signaling arose from single-cell studies and was later verified by work on lobule preparations in which the normal microscopic structure of the gland was preserved.^2^ Recently, Takahiro Takano and David Yule have carried these studies further by examining Ca^2+^ signal generation in pancreatic acinar cells in the intact pancreas in the living mouse.^6^ This in vivo study showed that physiological activation, either by electrical stimulation of the vagal nerve, infusion of cholecystokinin (CCK) or intake of a meal, elicited Ca^2+^ signals in the acinar cells. At low levels of stimulation, these signals were predominantly local, confined to the apical granule-containing region, whereas at higher intensities of stimulation, the signals became globalized.^6^ Thus, this new in vivo study confirmed, in a real physiological setting, earlier work on isolated cells and pancreatic lobules.^2^ An important new and unexpected finding from the in vivo study was the appearance of low level Ca^2+^ signaling events, even in the complete absence of external stimulation, in a sub-population of acinar cells. This was due to the endogenous basal CCK level in the blood, since it was abolished by a CCK antagonist, but not by atropine.^6^

The integrative neuroscientists have frequently been more advanced technically than other physiologists and have perhaps also been particularly concerned about studying physiologically relevant situations. Thus, already more than 15 yr ago, Carl Petersen and his group showed that the cortical representation of the physiologically important whisker touch could be studied by voltage-sensitive dye imaging in freely moving mice.^7^ Technically, this proved the usefulness of fiber optics to image cortical sensory activity with high resolution and, importantly, the results of these studies demonstrated differential processing of sensory input depending on behavior.^7,8^

In a very recent study of neurons and astrocytes in awake and behaving mice, Alex Verkhratsky and collaborators have shown that locomotion induces fundamentally different patterns of Ca^2+^ signaling in astrocytes and neurons.^9^ Whereas the neuronal Ca^2+^ concentration increased almost immediately after the onset of locomotion and faithfully followed such episodes, the astrocytic Ca^2+^ signals were delayed by several seconds, developing on a different timescale.^9^ These findings provide important fresh evidence for the special and distinct roles played by the astrocytes in sustaining neurons and supporting their function.^9,10^

These are just a few examples of findings that could only have been made by studies paying close attention to the establishment of real physiological conditions. When I started out in research, more than 50 yr ago, we generally did work in vivo under proper physiological conditions, but we did not have tools to get mechanistic information about cellular control mechanisms. This became possible in single-cell studies carried out in the 1980s, 1990s, and 2000s. Now, we have tools that allow us to observe molecular events simultaneously in multiple cell types in intact tissues and increasingly in living behaving animals. In the future, we may perhaps be able to do some of these studies safely in humans and might then also carry out investigations under critical pathological conditions. This would open up amazing new vistas of great consequence for diagnosis and treatment.

## Data Availability

There are no data in this editorial.
